# Escape from natural enemies depends on the enemies, the invader, and competition

**DOI:** 10.1002/ece3.6737

**Published:** 2020-09-11

**Authors:** Jacob E. Lucero, Nafiseh Mahdavi Arab, Sebastian T. Meyer, Robert W. Pal, Rebecca A. Fletcher, David U. Nagy, Ragan M. Callaway, Wolfgang W. Weisser

**Affiliations:** ^1^ Department of Biology York University Toronto ON Canada; ^2^ Terrestrial Ecology Research Group Department of Ecology and Ecosystem Management School of Life Sciences Weihenstephan Technical University of Munich Freising Germany; ^3^ Department of Biological Sciences Montana Technological University Butte MT USA; ^4^ Institute of Biology Faculty of Sciences University of Pecs Pecs Hungary; ^5^ School of Plant and Environmental Sciences Virginia Tech Blacksburg VA USA; ^6^ Divison of Biological Sciences and the Institute on Ecosystems University of Montana Missoula MT USA

**Keywords:** biogeography, enemy release hypothesis, insect herbivory, invasive species, plant–herbivore interactions, plant–soil feedbacks, *Solidago canadensis*, *Tanacetum vulgare*

## Abstract

1. The enemy release hypothesis (ERH) attributes the success of some exotic plant species to reduced top‐down effects of natural enemies in the non‐native range relative to the native range. Many studies have tested this idea, but very few have considered the simultaneous effects of multiple kinds of enemies on more than one invasive species in both the native and non‐native ranges. Here, we examined the effects of two important groups of natural enemies–insect herbivores and soil biota–on the performance of *Tanacetum vulgare* (native to Europe but invasive in the USA) and *Solidago canadensis* (native to the USA but invasive in Europe) in their native and non‐native ranges, and in the presence and absence of competition.
2. In the field, we replicated full‐factorial experiments that crossed insecticide, *T. vulgare–S. canadensis* competition, and biogeographic range (Europe vs. USA) treatments. In greenhouses, we replicated full‐factorial experiments that crossed soil sterilization, plant–soil feedback, and biogeographic range treatments. We evaluated the effects of experimental treatments on *T. vulgare* and *S. canadensis* biomass.
3. The effects of natural enemies were idiosyncratic. In the non‐native range and relative to populations in the native range, *T. vulgare* escaped the negative effects of insect herbivores but not soil biota, depending upon the presence of *S. canadensis*; and *S. canadensis* escaped the negative effects of soil biota but not insect herbivores, regardless of competition. Thus, biogeographic escape from natural enemies depended upon the enemies, the invader, and competition.

*Synthesis:* By explicitly testing the ERH in terms of more than one kind of enemy, more than one invader, and more than one continent, this study enhances our nuanced perspective of how natural enemies can influence the performance of invasive species in their native and non‐native ranges.

## Graphical abstract

We examined a leading hypothesis of plant invasion, the enemy release hypothesis, in terms of multiple invasive species, multiple natural enemies, and multiple continents. We found that escape from natural enemies depends on the enemies, the invader, and competition.

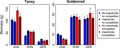

## 1 INTRODUCTION

The enemy release hypothesis (ERH) is a leading explanation for successful biological invasions by exotic plant species. The ERH asserts that biogeographic translocation allows some exotic plant species to leave behind their natural enemies, resulting in relative freedom from top‐down controls in non‐native communities relative to native communities (Keane & Crawley, 2002). This idea can be tested experimentally by excluding natural enemies in the native and non‐native ranges of invaders (Maron & Vilà, 2001). The ERH predicts that enemy exclusion should increase the performance of invaders to a greater extent in their native range (where enemies should have relatively strong top‐down effects) than in their non‐native range (where enemies should have relatively weak top‐down effects) (Keane & Crawley, 2002; Maron & Vilà, 2001). The ERH has attracted considerable empirical attention (see reviews by Jeschke et al., 2012; Liu & Stiling, 2006), but relatively few studies have employed the experimental, biogeographically explicit approach outlined above. Also, most studies have examined the ERH in the context of specialist herbivores, but enemy release from several kinds of enemies has been demonstrated, including antagonistic soil biota (Maron, Klironomos, Waller, & Callaway, 2014; Reinhart, Packer, van der Putten, & Clay, 2003), aboveground fungal pathogens (DeWalt, Denslow, & Ickes, 2004), interspecific competitors (Callaway et al., 2011), generalist herbivores (Vermeij, Smith, Dailer, & Smith, 2009), and even postdispersal seed predators (Lucero et al., 2019).

Of the biogeographically explicit tests of the ERH, very few have excluded multiple kinds of natural enemies simultaneously or in factorial experiments (but see DeWalt et al., 2004; Williams, Auge, & Maron, 2010). This is an important knowledge gap because plants function in complex biotic environments where they may be attacked by different kinds of natural enemies at once, including competitors, herbivores, and pathogens (Cipollini, 2004; DeLong, Fry, Veen, & Kardol, 2019; Fernandez‐Conradi et al., 2018). Importantly, the effects of these enemies are not necessarily additive (reviewed by Stephens, Srivastava, & Myers, 2013). For instance, DeWalt et al. (2004) excluded fungal pathogens and insect herbivores from populations of the invasive shrub *Clidemia hirta* in its native and non‐native ranges. In understory sites in the native range, survival of *C. hirta* increased by 19% when treated with fungicide, 12% when treated with insecticide, and 41% when treated with both. Pesticide applications did not affect *C. hirta* survival in the non‐native range. Taken together, these findings suggest that antagonistic fungi and insect herbivores acted singly and jointly (in a nonlinear fashion) to limit *C. hirta* survival in the native range but not in the non‐native range, as predicted by the ERH. This example illustrates how testing the ERH on single enemy guilds in isolation limits our understanding of the effects of natural enemies on invasion trajectories, which in turn hinders our ability to explain, predict, and manage the spread of invasive species. These issues have crucial implications for the function of contemporary ecosystems (Bellard, Leroy, Thuller, Rysman, & Courchamp, 2016; Vilà et al., 2011) and economies (Pimentel, Zuniga, & Morrison, 2005; Seebens et al., 2019). Thus, biogeographic tests of the ERH that exclude multiple guilds of natural enemies simultaneously are sorely needed, as emphasized in the review of Roy, Lawson Handley, Schonrogge, Poland, and Purse (2011).

Here, we examined the effects of two important kinds of enemies–insect herbivores and antagonistic soil biota–on the performance of two invasive plant species–common tansy (*Tanacetum vulgare*; “tansy” hereafter) and Canada goldenrod (*Solidago canadensis*; “goldenrod” hereafter)–in their native and non‐native ranges. Specifically, we tested the key prediction derived from the ERH that excluding these enemies would improve the performance of tansy and goldenrod more in their respective native ranges than in their non‐native ranges. To do this, we replicated enemy exclusion experiments in Europe (where tansy is native but goldenrod is exotic and invasive) and in the USA (where goldenrod is native but tansy is exotic and invasive), using field‐based and greenhouse approaches. In Europe and the USA, tansy and goldenrod commonly co‐occur and may compete directly for limiting resources (Schittko, Runge, Strepp, Wolff, & Wurst, 2016; Werner, Brandbury, & Gross, 1980) or indirectly via associational effects (Hahn & Orrock, 2016; Kim & Underwood, 2015) and apparent competition (Orrock, Witter, & Reichman, 2008). Accordingly, our field experiments crossed insect exclusion treatments with interspecific competition treatments to account for the possibility that herbivory and competition interacted to influence plant performance.

## 2 MATERIALS AND METHODS

### 2.1 Study species

Tansy is native to Europe but was introduced to North America in the 17th century for medicinal and ornamental purposes and is now broadly distributed across this non‐native range (Mitich, 1995). In the non‐native range, some state governments have listed tansy as a noxious weed due to negative impacts on local biodiversity and pasture quality (LeCain & Sheley, 2014). In its native range, tansy is attacked by over 169 species of herbivorous insects, including 29 specialist species, which can substantially decrease performance (Kleine & Müller, 2014). Little is known about the effects of insect herbivory in the non‐native range. In addition, to our knowledge, no study has contrasted the effects of soil biota on tansy performance in native versus non‐native ranges.

Goldenrod is native to North America but was introduced to Europe in the 17th century, also for medicinal and ornamental purposes. Since its introduction to Europe, goldenrod has become one of the most abundant and problematic invasive plant species on the continent (Rebele, 2000). In non‐native ranges, goldenrod invasion reduces the biodiversity of native plant and animal communities (Ledger et al., 2015; Lenda et al., 2019; Skorka, Lenda, & Tryjanowski, 2010), alters soil biogeochemical processes (Lu, Qiu, Chen, & Li, 2005), and disrupts interactions with pollinators (Fenesi et al., 2015). Despite these negative effects, goldenrod in Europe has reportedly facilitated the growth of tansy (Schittko & Wurst, 2014), perhaps by suppressing antagonistic soil biota (Zhang, Jin, Tang, & Chen, 2009). In its native range, goldenrod is a strong competitor but does not reduce community‐level biodiversity as it does in the non‐native range (Ledger et al., 2015; but see Carson & Root, 1999, 2000). In the native range, goldenrod is attacked by a rich community of insect herbivores, which can decrease growth, fecundity and competitive ability (Cain, Carson, & Root, 1991; Carson & Root, 1999, 2000; Long, Mohler, & Carson, 2003). In contrast, goldenrod is attacked by a relatively depauperate generalist insect community in the non‐native range (Jobin, Schaffner, & Nentwig, 1996), and the effects of insect herbivory on goldenrod performance in the non‐native range are unclear. In addition, as with tansy, the extent to which soil biota affects goldenrod performance in native versus non‐native ranges has received little attention.

### 2.2 Study area

We examined the effects of excluding natural enemies (i.e., insect herbivores and soil biota) on the performance of tansy and goldenrod using field and greenhouse experiments replicated in Europe (the native range of tansy and the non‐native range of goldenrod) and North America (the native range of goldenrod and the non‐native range of tansy). Experiments in Europe were conducted in Germany and/or Hungary, and experiments in North America were conducted in Montana, USA. Plants used in our experiments were derived from seeds collected in 2009 by hand from wild populations in Germany, Hungary, and Montana (see Appendix S1: Table S1 for locations). Tansy and goldenrod plants co‐occurred at all seed collection sites.

### 2.3 Field experiments

We replicated full‐factorial field experiments in Germany, Hungary, and Montana to contrast the individual and joint effects of interspecific competition and insect herbivory on the performance of tansy and goldenrod in their native and non‐native ranges. Two sites were used in Europe to increase the scope of inference in the native range. In each country, we established one experimental garden (see Appendix S1: Table S2 for locations and climatic information), which were fenced to exclude mammalian herbivores. Wild populations of tansy and goldenrod were in the vicinity (<1 km) of each experimental garden. Experimental gardens in Germany and Hungary were comprised of 15 experimental blocks, each consisting of six, 50 × 50‐cm plots with a 50‐cm walking path around each block. We randomly assigned one of the following planting treatments to each plot: (a) a monoculture of common tansy (6 plants), (b) a monoculture of goldenrod (6 plants), or (c) a biculture of common tansy and goldenrod (3 + 3 plants), with each planting treatment replicated twice per block. Thus, total plant density was held constant in each plot (6 plants), and interspecific competition occurred only in the biculture plots. The experimental garden in Montana was comprised of six experimental blocks, each consisting of 15 plots that received the same randomized planting treatments listed above, with each treatment replicated five times per block. In sum, gardens in all countries consisted of 90 plots that were distributed randomly over blocks with respect to planting treatment, and the same treatments with the same number of replicates were realized in all gardens.

Tansy and goldenrod were transplanted into experimental gardens from plants started as seeds in the greenhouse. In April 2010, tansy and goldenrod seeds (see Table S1 for seed sources) were sown into 200 ml pots in a 1:1 mixture of sand and potting soil and kept in a naturally lit greenhouse (see Appendix S1: Table S3 for greenhouse locations). After germination, seedlings were thinned down to one individual per pot and watered once daily. In June 2010, plants were transplanted to experimental plots in two rows of three (i.e., six plants per plot, as described above), spaced 10 cm apart. In biculture plots, plant identity alternated every other plant.

In each experimental garden, we randomly selected half of the plots assigned to each planting treatment to be treated with insecticide. Specifically, we treated 3 out of 6 plots per block in Germany and Hungary, and 6 or 9 (*n* = 3 of each) out of 15 plots per block in Montana. Thus, 45 out of 90 plots in each garden were treated with insecticide. We used a mixture of the systemic insecticide Biscaya^®^ (active ingredient *thiacloprid*, (Z)‐3‐(6‐chloro‐3‐pyridylmethyl)‐1, 3‐thiazolidin‐2‐ylidenecyanamide) and the knock‐down insecticide Decis^®^ (active ingredient *deltamethrin*, (S)‐cyano‐3‐pehoxybenzyl(1R)‐cis‐3‐(2,2‐dibromovinyl)‐2,2‐dimethyl‐cyclo‐propanecarboxylate). Neither insecticide is selective with respect to taxa. Insecticides were diluted with water as per label instructions and were applied using a nonmotorized backpack sprayer. We applied insecticides in June and August, 2010, and in May, June, July, and August 2011. On application days, plots not selected for insecticide treatment were sprayed with an equal volume of water. We protected nontarget plants from insecticide drift by spraying only on windless days, taking great care to spray only target plants.

In September 2011, herbivore damage on experimental plants was assessed, the aboveground biomass of each plant was measured, and the effects of insect herbivory and plant–plant competition were calculated. We reported herbivore damage as the number of leaves on the tallest shoot of each plant with visible signs of insect damage (chewed holes or leaf mines). This measure did not account for the size of plants and was not used to detect patterns of enemy release because herbivore damage per se may not translate to effects on plant performance (biomass in this case). Rather, we evaluated herbivore damage to verify that insecticide applications worked as expected. Immediately after herbivore damage was assessed, we harvested the aboveground biomass of all plants and weighed each plant individually after drying plants at 70°C for 48 hr to constant mass. We calculated the effects of insect herbivores on plant biomass by contrasting the mean biomass of plants treated versus not treated with insecticides. We calculated the effects of interspecific competition by contrasting the mean biomass of plants grown in monocultures versus bicultures. Biomass is likely a good proxy for fitness in our system because both tansy and goldenrod rely extensively on vegetative reproduction to spread and impact local communities (Dong, Lu, Zhang, Chen, & Li, 2006; Mitich, 1995), and biomass for both species closely predicts investment in sexual reproduction (Hartnett, 1990). We harvested aboveground biomass to leave rhizospheres intact to facilitate soil harvesting for greenhouse experiments.

### 2.4 Greenhouse experiments

We replicated full‐factorial greenhouse experiments in Hungary and Montana to examine the effects of local soil biota on the performance of tansy and goldenrod in their native and non‐native ranges (see Table S3 for greenhouse locations and growing conditions). At the conclusion of the field experiments described above, we collected 1 L of soil from each plot that had been occupied by monocultures of either tansy or goldenrod in Hungary and the USA (*n* = 30 soil samples per species per country). Soil samples from each plot were kept separate and were not pooled. One half liter (0.5 L) of each soil sample was sterilized by autoclaving for two, 60‐min intervals with a 24‐hr rest between intervals. The other 0.5 L of each soil sample was left unsterilized so that the soil biota remained intact (i.e., “live”). We then filled 250 ml pots with either sterilized or live soil. We planted one seed per species (seeds were derived from the same sources as reported above; see Appendix S1: Table S1) into a total of 240 pots (*n* = 60 per species per soil source) in each greenhouse. After 30 days, seedlings were repotted into 500 ml pots. After 90 days in 500 ml pots, plants were harvested (15 July 2011), soil was gently washed away from roots, and total (i.e., above‐ and belowground) biomass was recorded for each plant, using the same drying and weighing protocol described above. The effects of soil biota were inferred by contrasting the total biomass of plants in sterilized versus live soils. Importantly, our sterilization treatments excluded soil mutualists and antagonists simultaneously. Thus, any effects of soil sterilization on plant performance reflected the net positive and negative effects of beneficial and antagonistic soil biota combined.

### 2.5 Statistical analyses

We used linear mixed‐effects models to evaluate the effectiveness of insecticide applications on experimental plants in the field. Our models used herbivore damage (averaged across individuals per plot) as the response variable; biogeographic range (i.e., whether interactions took place in the target species’ native or non‐native range), country in Europe (Germany or Hungary), insecticide treatment, and competition treatment as interacting fixed factors; and experimental block as a random factor. We analyzed tansy and goldenrod with independent models. If insecticide treatments worked as expected, herbivore damage should be greatest on untreated plants, resulting in a significant main effect of insecticide for both species.

We used linear mixed‐effects models to evaluate the independent and joint effects of biogeographic range, insect herbivory, and competition on plant performance (i.e., aboveground biomass). Our models used aboveground biomass of individual plants as the response variable; biogeographic range, country in Europe, insecticide treatment, and competition treatment as fixed factors; and experimental plot nested in block as a random factor. Tansy and goldenrod were analyzed with independent models, and for the analysis of each species, we only used the plots of each block that contained the target species. If the effects of insect herbivores follow predictions derived from the ERH, herbivore exclusion (i.e., insecticide application) should increase the performance of focal invaders to a greater extent in the native range than in the non‐native range. Specifically, herbivore exclusion in Europe should improve the performance of tansy to a greater degree than herbivore exclusion in the USA, and herbivore exclusion in the USA should improve the performance of goldenrod to a greater degree than herbivore exclusion Europe, resulting in significant insecticide × biogeographic range interactions.

We used linear models to evaluate the independent and joint effects of biogeographic range, soil biota, and soil‐mediated plant–plant interactions on performance (i.e., total biomass) in greenhouse experiments. Our models used total biomass as the response variable; and biogeographic range, sterilization treatment (sterilized vs. live soils), and soil source (soils cultured by conspecifics vs. heterospecifics) as interacting fixed factors. We analyzed tansy and goldenrod independently. If the effects of soil biota follow predictions derived from the ERH, sterilizing soils (i.e., excluding antagonistic soil biota) should increase the performance of invaders in their native range to a greater extent than in their non‐native range. Specifically, soil sterilization in Hungary should increase the performance of tansy to a greater degree than soil sterilization in the USA; and soil sterilization in the USA should increase the performance of goldenrod to a greater degree than soil sterilization in Hungary, resulting in significant sterilization × biogeographic range interactions.

All analyses were performed in R version 3.6.0 (R Development Core Team, 2018). We used the base “lm” function for linear models and the “lme” function of the “nlme” package version 3.1‐139 (Pinheiro, Bates, DebRoy, & Sarkar, 2016) for linear mixed‐effects models. Full models were reduced in a stepwise, backwards variable selection removing the least significant term until minimum adequate models were reached. Data were cube‐root or log‐transformed to improve normality (see Table 1, Appendix S1: Table S4). We calculated contrasts for specific combinations of treatments using the “contrast” function from the “contrast” package version 0.21 (Kuhn, Weston, Wing, Forester, & Thaler, 2016). For models on transformed data, back‐transformed contrast predictions and standard errors (*SE*s) were calculated. Due to back transformation, *SE*s of contrasts were asymmetrical and are thus reported as values ± one *SE*. Stated *t‐* and *p‐*values are taken directly from the calculated contrasts from back‐transformed values.

**TABLE 1:** Results of field experiments replicated in Germany, Hungary, and the USA that assessed the individual and joint effects of biogeographic range (native vs. non‐native), insect exclusion (insecticide application), interspecific competition, and country within Europe (Hungary vs. Germany) on the performance of tansy and goldenrod, according to linear mixed‐effects models with aboveground biomass as the response variable; biogeographic range, insecticide treatment, competition treatment, and country as interacting fixed factors; and experimental plot nested in block (not shown) as a random factor

Explanatory variable	Species	
	Tansy	Goldenrod
Range	***F*_1,33_ = 101; *p ***<< **.001**	***F*_1,34_ = 114; *p ***<< **.001**
Insecticide	***F*_1,139_ = 5.24; *p* = .024**	(*F*_1,140_ = 0.64; *p* = .425)^8^
Competition	***F*_1,139_ = 5.83; *p* = .017**	*F*_1,140_ = 2.92; *p* = .090
Country (within Europe)	***F*_1,33_ = 52.5; *p ***<< **.001**	(*F*_1,33_ = 0.01; *p = *.927)^7^
Range × insecticide	***F*_1,139_ = 10.2; *p* = .002**	(*F*_1,137_ = 0.16; *p* = .687)^4^
Range × competition	***F*_1,139_ = 4.15; *p* = .044**	***F*_1,140_ = 8.00; *p* = .005**
Insecticide × competition	***F*_1,139_ = 5.19; *p* = .024**	(*F*_1,138_ = 0.78; *p* = .378)^5^
Country × insecticide	(*F*_1,137_ = 0.06; *p* = .813)^3^	(*F*_1,136_ = 0.05; *p* = .826)^3^
Country × competition	(*F*_1,138_ = 0.90; *p* = .344)^4^	(*F*_1,139_ = 1.15; *p* = .286)^6^
Range × insecticide ×competition	(*F*_1,136_ = 0.60; *p* = .442)^2^	(*F*_1,135_ = 0.52; *p* = .471)^2^
Country × insecticide ×competition	(*F*_1,135_ = 0.09; *p* = .766)^1^	(*F*_1,134_ = 0.06; *p* = .815)^1^

Note

Significant (i.e., *p* ≤ .05) effects appear in bold. Numerical superscripts indicate the order in which nonsignificant terms (given in parentheses) were removed from the final model. Tansy and goldenrod were evaluated with independent models.

Data were cube‐root transformed to improve normality.

## 3 RESULTS

### 3.1 Field experiments

#### 3.1.1 Tansy

Insecticide treatments reduced herbivore damage on tansy across all ranges and treatments, but more so in the native range. Herbivore damage was much greater in Hungary than any other country (Appendix S1: Figure S1), but insect herbivores in all countries generally damaged more leaves on untreated plants than treated plants, regardless of interspecific competition (Appendix S1: Figure S1). Thus, insecticide treatments worked as expected. These findings corresponded to significant main effects of biogeographic range, insecticide treatment, and country within Europe; and significant biogeographic range × insecticide treatment and country within Europe × insecticide treatment interactions (Appendix S1: Table S4). Importantly, however, herbivore damage did not necessarily translate to effects on biomass (Table 1).

**FIGURE 1:**
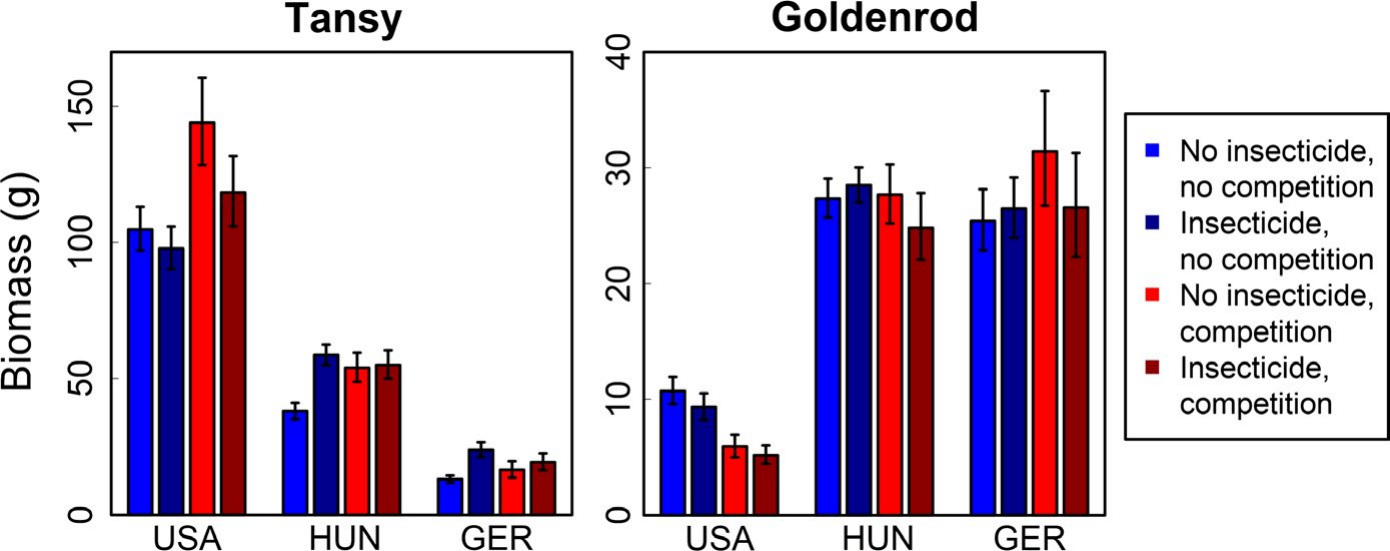
Means ± *SE* for aboveground biomass of tansy and goldenrod (per plant) in field experiments replicated in the USA, Hungary (HUN), and Germany (GER) that crossed herbivore exclusion (i.e., insecticide application) and interspecific competition treatments. Note the different scales of *y*‐axes for each plant species

The effects of insect exclusion on the aboveground biomass of tansy depended upon biogeographic context and the presence of goldenrod (Figure 1). Consistent with predictions derived from the ERH, insect exclusion in the absence of goldenrod increased the biomass of tansy in the native range (Hungary and Germany) by at least 10.4 g (±*SE*: 7.0, 14.2; *t*_781_ = 3.5, *p* < .001) but had no effect in the non‐native range (USA; *t*_781_=−0.23, *p* = .822). However, we observed a different pattern in the presence of goldenrod. When goldenrod was present, insect exclusion had no effect on the biomass of tansy in any country. Thus, insect herbivores reduced tansy performance in the native range only when goldenrod was absent, but had no effects in the non‐native range. In addition, regardless of experimental treatments, tansy grew at least 63.9 g (±*SE*: 51.6, 77.3; *t*_783_ = 6.39; *p* < .001) larger in the USA than in either European country, and tansy in Hungary grew 32.8 g (±*SE*: 26.9, 39.2; *t*_783_ = 7.29, *p* < .001) larger than tansy in Germany. These trends corresponded to significant main effects of biogeographic range, insecticide treatment, and country within Europe; and significant biogeographic range × insecticide treatment and insecticide treatment × competition interactions (Table 1).

The effects of goldenrod on tansy biomass depended upon insecticide treatment and biogeographic range (Figure 1). When insecticides were applied, tansy achieved the same biomass in the presence and absence of goldenrod, in all countries. However, without insecticides, tansy in all countries grew larger in the presence of goldenrod than in its absence (Germany: 4.67 ± *SE*: 7.34, 2.27; Hungary: 9.78 ± *SE*: 15.1, 4.82; USA: 44.9 ± *SE* 58.6, 32.0; all *t*_783_ ≥ 2.05, *p* ≤ .04). Thus, regardless of biogeographic context, goldenrod had neutral effects on the biomass of tansy when insects were excluded, but goldenrod facilitated the biomass of tansy when insects were present. These trends corresponded to a significant main effect of competition and a significant insecticide treatment × competition interaction (Table 1).

#### 3.1.2 Goldenrod

Insecticide treatments on goldenrod generally reduced herbivore damage, and more so in the native range. As with tansy, herbivore damage was much greater in Hungary than any other country (Appendix S1: Figure S1), but insect herbivores in all countries generally damaged more leaves on untreated plants than treated plants, regardless of interspecific competition (Appendix S1: Figure S1). These trends corresponded to significant main effects of biogeographic range, insecticide, and country within Europe; and significant biogeographic range × insecticide treatment and country within Europe × insecticide treatment interactions (Appendix S1: Table S4). Importantly, however, herbivore damage did not translate to effects on biomass (Table 1).

Insect exclusion had no effect on the aboveground biomass of goldenrod, regardless of biogeographic range or the presence of tansy (Figure 1). Contrary to predictions derived from the ERH, insect exclusion did not increase the biomass of goldenrod in either the native (USA) or non‐native (Hungary and Germany) range, regardless of the presence of tansy (Table 1). Importantly, however, goldenrod grew on average 19.6 g (±*SE*: 17.1, 22.3; *t*_759_ = 11.0; *p* < .001) larger in Hungary and Germany than in the USA, with similar biomass in Hungary and Germany (Table 1). These trends corresponded to a significant main effect of biogeographic range, but no significant interactions involving biogeographic range or insecticide application (Table 1).

The effects of tansy on goldenrod biomass depended upon biogeographic range (Figure 1). In the USA, goldenrod grew 4.4 g (±*SE*: 2.9, 6.1; *t*_759_ = 3.3, *p* = .001) larger in the absence of tansy than in its presence, suggesting competition, but in the non‐native ranges of Hungary and Germany, goldenrod achieved the same biomass whether or not common tansy was present (*t*_759_=−0.23, *p* = .817). This pattern was unaffected by insect exclusion. Thus, common tansy imposed competitive effects on goldenrod in the native range but not in Hungary or Germany, regardless of insect exclusion. These findings corresponded to a significant biogeographic range × competition interaction (Table 1).

### 3.2 Greenhouse experiments

#### 3.2.1 Tansy

**FIGURE 2:**
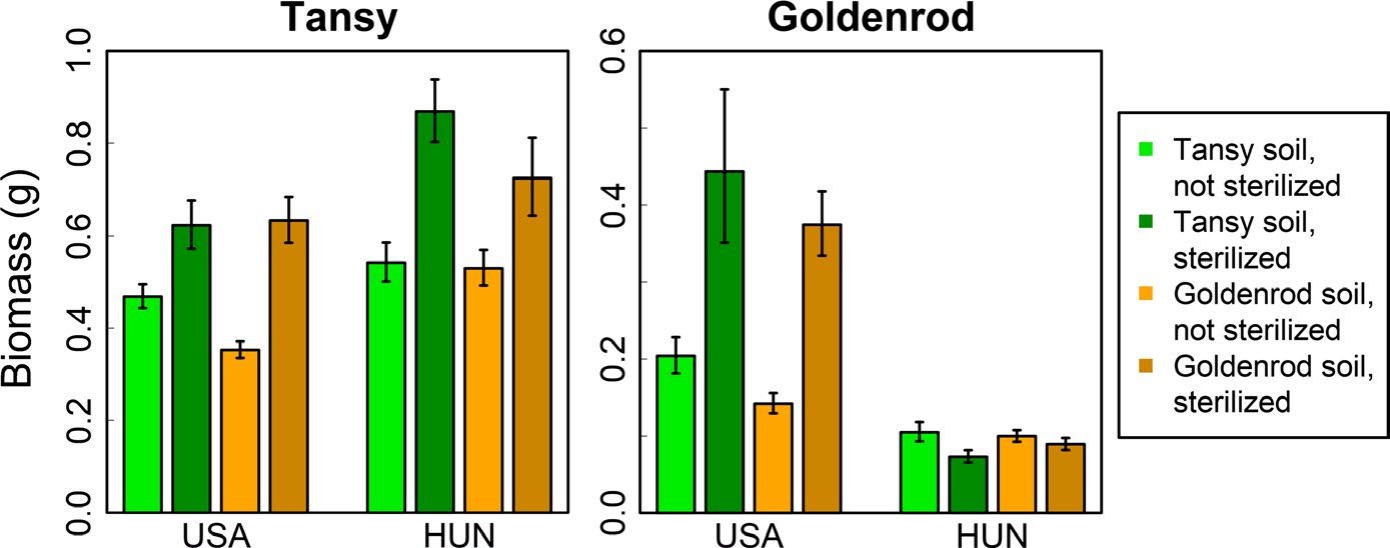
Mean ± *SE* for total biomass of tansy and goldenrod (per plant) grown in sterilized and unsterilized soils cultured by either conspecifics or heterospecifics in greenhouse experiments replicated in Hungary (HUN) and the USA. Note the different scales of *y*‐axes for each plant species

For tansy biomass, the effects of suppressing soil biota (soil sterilization) did not depend upon biogeographic range or soil source–whether soils were cultured by conspecifics or heterospecifics (Figure 2). Contrary to predictions derived from the ERH, suppressing soil biota increased tansy biomass in both the native and non‐native range, regardless of soil source (Hungary: 0.26 ± *SE*: 0.29, 0.23; USA: 0.22 ± *SE*: 0.25, 0.19; all *t*_215_ ≥ 6.92, *p* < .001). In addition, across all treatments, tansy grew 0.15 g (±SE: 0.11, 0.18; *t*_215_ ≥ 4.25, *p* < .001) larger in the native range (Hungary) than the non‐native range (USA). These findings corresponded to significant main effects of biogeographic range, soil source, and sterilization treatment, but no significant sterilization treatment × biogeographic range interaction (Table 2).

**TABLE 2:** Results of greenhouse experiments replicated in Hungary and the USA that evaluated the effects of soil biota conditioned by conspecifics or heterospecifics on the performance of tansy and goldenrod, according to a linear model with total biomass as the response variable, biogeographic range (native vs. non‐native), soil source (conspecific vs. heterospecific), and soil sterilization treatment (sterilized vs. nonsterilized) as interacting fixed factors, with pot replicate nested within block (i.e., soil source) (not shown) as a random factor

Explanatory variable	Species	
	Tansy	Goldenrod
Range	***F*_1,215_ = 18.3; *p ***<<** .001**	***F*_1,188_ = 152; *p* **<<** .001**
Soil source	***F*_1,215_ = 3.89; *p* = .050**	(*F*_1,187_ = 0.45; *p* = .501)^4^
Sterilization	***F*_1,215_ = 47.9; *p ***<<** .001**	***F*_1,188_ = 9.80; *p* = .002**
Range × soil source	(*F*_1,213_ < 0.01; *p* = .953)^3^	(*F*_1,186_ = 3.67; *p* = .057)^3^
Range × sterilization	(*F*_1,212_ < 0.01; *p = *.989)^2^	***F*_1,188_ = 51.7; *p ***<< **.001**
Soil source × sterilization	(*F*_1,214_ = 0.04; *p* = .842)^4^	(*F*_1,185_ = 1.23; *p* = .269)^2^
Range × soil source × sterilization	(*F*_1,211_ = 3.54; *p* = .061)^1^	(*F*_1,184_ = 0.03; *p* = .858)^1^

Note

Significant (i.e., *p* ≤ .05) effects appear in bold. Numerical superscripts indicate the order in which nonsignificant terms (given in parentheses) were removed from the final model. Tansy and goldenrod were evaluated with independent models.

Data were cube‐root transformed to improve normality.

#### 3.2.2 Goldenrod

For goldenrod biomass, the effects of soil sterilization depended upon biogeographic range (Figure 2). Consistent with predictions derived from the ERH, suppressing soil biota increased goldenrod biomass in the native range only, regardless of soil source (Hungary: −0.02 ± *SE* −0.01, −0.03; *t*_188_=−1.91, *p* = .058; USA: 0.23 ± *SE*: 0.25, 0.21; all *t*_188_ = 7.61, *p* < .001). Thus, antagonistic biota in both conspecific and tansy soils reduced goldenrod biomass in the native range, but had no significant effects in the non‐native range. In addition, across all treatments, goldenrod grew 0.18g (±*SE*: 0.16, 0.20; *t*_188_ = 12.95, *p* < .001) larger in the native range (USA) than the non‐native range (Hungary). These findings corresponded to significant main effects of biogeographic range and sterilization treatment, and a significant biogeographic range × sterilization treatment interaction (Table 2).

## 4 DISCUSSION

A handful of studies have experimentally shown that natural enemies inhibit invasive plant species more in their native ranges than in their non‐native ranges (e.g., DeWalt et al., 2004; Lucero et al., 2019; Williams et al., 2010), but we examined the effects of multiple enemy guilds on more than one invasive species in both ranges. We found that enemy release from insect herbivores, but not antagonistic soil biota, and antagonistic soil biota, but not insect herbivores, may enhance the performance of tansy and goldenrod, respectively, in their non‐native ranges relative to their native ranges. These findings suggest that biogeographic escape from effects natural enemies can depend upon the enemies, the invader, and competition.

Our study emphasizes the importance of testing the ERH by contrasting the effects of natural enemies in the native versus non‐native ranges of invasive species (Keane & Crawley, 2002; Maron & Vilà, 2001). To date, most empirical examinations of the ERH have conducted biogeographic comparisons of *enemy loads* and inferred enemy release when fewer enemy species attacked focal invaders in the non‐native range compared to the native range (see reviews by Meijer, Schilthuizen, Beukeboom, & Smit, 2016; Roy et al., 2011). This approach may demonstrate escape from certain natural enemies (e.g., Mitchell & Power, 2003), but it does not show release because reduced enemy loads may not translate to increased abundance or performance for the invader (Beckstead & Parker, 2003). To this point, previous work has shown that goldenrod is attacked by more species of insect herbivores in the native range than the non‐native range (Jobin et al., 1996; Long et al., 2003), but our experiments found no evidence that this pattern translated to plant performance in the field–insects damaged goldenrod in both native and non‐native ranges (Appendix S1: Figure S1) but failed to reduce biomass in either range (Figure 1; see also van Kleunen & Schmid, 2003). Furthermore, herbivore damage on tansy was by far greatest in Hungary (Appendix S1: Figure S1), but plants in Hungary outgrew plants in Germany (Figure 1).

Our field experiments suggested that associational effects between common tansy and goldenrod can influence patterns of enemy release. Associational effects arise when herbivore effects on a focal plant change due to the presence or identity of neighboring plants (reviewed by Barbosa et al., 2009; Callaway, 2007; Underwood, Inouye, & Hamback, 2014). In this context, insect herbivory reduced tansy biomass in the native range of Europe only when goldenrod was absent, whereas insect herbivory had no effect on tansy in the USA, regardless of the presence of goldenrod (Figure 1). Said differently, in Europe only, the presence of goldenrod at least partially ameliorated the negative effects of insect herbivory on tansy biomass. Biogeographic differences in associational effects relevant to plant invasion have been hypothesized (Orrock, Holt, & Baskett, 2010), but to our knowledge, this is the first empirical evidence that the outcome of associational effects can depend upon whether interactions occurred in the native or non‐native range of a focal species. Importantly, our interpretation of associational effects should be viewed with some caution because at the plot level, we controlled for total plant density but not species‐specific frequency, which can influence the detection of associational effects (Underwood et al., 2014). Regardless, our findings underscore a growing body of research suggesting that invasive species can impose associational effects on native neighbors for better (Atwater, Bauer, & Callaway, 2011; van Ruijven, De Deyn, & Berendse, 2003) or for worse (Beckstead, Meyer, & Ausperger, 2008; Enge, Nylund, & Pavia, 2013; Orrock et al., 2008).

Our study agrees with other reports that goldenrod can facilitate tansy. Goldenrod in Europe is generally associated with negative effects on biodiversity at the community level, but Schittko and Wurst (2014) showed that for tansy in Europe, spatial association with goldenrod can increase growth. Our field experiments expanded these findings to the USA, but there only when insects were present (Figure 1). Zhang et al. (2009) suggested that goldenrod could facilitate tansy by suppressing antagonistic soil biota, but we found no evidence for this because goldenrod soils in both Hungary and the USA suppressed tansy performance (Figure 2). However, plant–soil feedbacks themselves are highly conditional (DeLong et al., 2019), with feedbacks for the congener *S. gigantea* varying twofold to 10‐fold among sites and among source populations (Maron, Luo, Callaway, & Pal, 2015). With respect to our field experiments, we speculate that in the USA, goldenrod facilitated tansy primarily by providing associational resistance against insect herbivory. Importantly, plant–plant facilitation can occur via a number of nonmutually exclusive mechanisms, which include associational effects (reviewed by Callaway, 2007; Filazzola & Lortie, 2014).

Our greenhouse experiments suggested that goldenrod in the non‐native range may escape the effects of antagonistic soil biota relative to populations in the native range. This is consistent with a broad literature showing that exotic invasive plant species tend to suffer much less inhibition from soil biota than do co‐occurring natives in plant–soil feedback experiments (Agrawal et al., 2005; Kardol, Cornips, Van Kempen, Bakx‐Schotman, & van der Putten, 2007; Klironomos, 2002; Kulmatiski, Beard, Stevens, & Cobbold, 2008; MacDougall, Rillig, & Klironomos, 2011; Pendergast, Burke, & Carson, 2013). In addition, biogeographic comparisons of plant–soil feedbacks have shown that some invasive species experience stronger negative soil feedbacks in their native ranges than their non‐native ranges (Callaway, Thelen, Rodriguez, & Holben, 2004; Maron et al., 2014; Reinhart & Callaway, 2006; Reinhart et al., 2003; Yang et al., 2013; Zuppinger‐Dingle et al., 2011), which is consistent with the ERH. However, we suggest caution in interpreting our results because the effects of soil sterilization in greenhouse conditions do not always translate to the field (Schittko et al., 2016). Regardless, our study suggests that escaping inhibition from soil biota (Figure 2) may help invasive populations of goldenrod to achieve greater biomass than native populations (Figure 1).

In field experiments, tansy and goldenrod both grew much larger in the non‐native range than in the native range, but this does not constitute strong support for the evolution of increased competitive ability (EICA) hypothesis (Blossey & Nötzold, 1995). Escape from natural enemies may permit translocated species to divert resources formerly required for defense toward growth and/or reproduction (Blossey & Nötzold, 1995). Over evolutionary time, this could result in a concomitant decrease in defensive characteristics and an increase in vigor (i.e., size or fecundity) for populations in the non‐native range relative to the native range (Blumental & Hufbauer, 2007; Maron, Vilà, & Arnason, 2004; Uesugi & Kessler, 2013). Empirical support for the EICA hypothesis is mixed, but there are many examples of invasive species performing better in the non‐native range relative to the native range (see reviews by Felker‐Quinn, Schweitzer, & Bailey, 2013; Jeschke et al., 2012; Lamarque, Delzon, & Lortie, 2011; Rotter & Holeski, 2018). In this context, tansy and goldenrod in the field both grew larger in non‐native ranges than native ranges, regardless of insect exclusion. This accords with reports that invasive populations can outperform those from the native range independently of the effects of natural enemies (Siemann, DeWalt, Zou, & Rogers, 2017). Such increased size in the non‐native range may help explain why tansy in field experiments only imposed significant competitive effects on goldenrod in the USA. For tansy, our greenhouse (i.e., common garden) experiments hinted that there may be a genetic (i.e., evolutionary) basis to biogeographic differences in plant size, but we did not control for founder effects because study plants came from single populations in each country. Thus, we cannot make strong inferences about the EICA hypothesis, despite our use of common gardens. Interestingly, we found that goldenrod in the greenhouse grew larger in the native range than the non‐native range, which is the opposite pattern predicted by the EICA hypothesis. This coincides with the report of Van Kleunen and Schmid (2003), which found no evidence for EICA in *S. canadensis*. However, Uesugi and Kessler (2013) found strong support for EICA in *S. altissima*, a closely related and functionally similar (Kabuce & Priede, 2010) congener.

As in many, if not all, biogeographic comparisons of populations of a species from native and non‐native ranges, it is very difficult to be certain if apples are being compared to apples. This is certainly true for goldenrod. *Solidago canadensis* is taxonomically complex. In North America, several taxonomic subunits have been recognized within the *S. canadensis* species complex, including *S. altissima*, which is considered to be a distinct species by some (Kabuce & Priede, 2010; Weber, 2001). Others have argued that the primary representative of the *Solidago* complex in Europe is closer to *S. altissima* than *S. canadensis* (Priedītis, 2002; but see Semple & Cook, 2006). Our study cannot speak to this debate because we collected seeds from single populations in each country, which precluded tests of population variation within any range (see Rosche et al., 2019).

It is well understood that natural enemies can have idiosyncratic effects on the performance of invasive plant species (Agrawal et al., 2005; Jeschke et al., 2012), but our study is unique in experimentally examining the effects of more than one kind of natural enemy on more than one invasive species in more than one range, simultaneously We found that the effects of natural enemies depended upon the identity of the focal invader, biogeographic context, and the focal invader's biotic environment–including the presence of heterospecific neighbors. These findings enhance our nuanced perspective of how natural enemies can influence the success of invasive plant species.

## CONFLICT OF INTEREST

The authors declare no conflicts of interest.

## AUTHOR CONTRIBUTION

**Jacob E. Lucero:** Visualization (supporting); Writing‐original draft (lead); Writing‐review & editing (equal). **Nafiseh Mahdavi Arab:** Conceptualization (equal); Investigation (lead); Methodology (supporting). **Sebastian T. Meyer:** Conceptualization (equal); Formal analysis (lead); Visualization (lead); Writing‐review & editing (equal). **Robert W. Pal:** Conceptualization (equal); Funding acquisition (supporting); Methodology (supporting); Project administration (supporting); Supervision (equal); Writing‐review & editing (equal). **Rebecca A. Fletcher:** Investigation (equal); Writing‐review & editing (equal). **David U. Nagy:** Conceptualization (equal); Investigation (equal); Project administration (equal); Supervision (equal); Writing‐review & editing (equal). **Ragan M. Callaway:** Conceptualization (equal); Funding acquisition (equal); Methodology (equal); Writing‐review & editing (lead). **Wolfgang W. Weisser:** Conceptualization (lead); Funding acquisition (lead); Methodology (equal); Project administration (lead); Supervision (equal); Writing‐review & editing (equal).

## Supporting information

Appendix S1

## Data Availability

Data for this article are publicly archived in the Dryad repository, https://doi.org/10.5061/dryad.hhmgqnkdt.
