# Supplementary material for: Escape from natural enemies depends on the enemies, the invader, and competition
Source: Ecol Evol. 2020 Sep 11;10(19):10818–28. doi: 10.1002/ece3.6737 (PMC7548199; doi:10.1002/ece3.6737)
Supplement: Supplementary file 1 — Appendix S1 [file ECE3-10-10818-s001.docx]

# **APPENDIX S1**

Appendix S1 for this article consists of one supporting figure: Fig. S1, and four supporting tables: Tables S1-4.

**Fig. S1.** Mean ± SE herbivore damage (per plant) on tansy and goldenrod in field experiments replicated in Germany (GER), Hungary (HUN), and the USA that crossed herbivore exclusion (i.e., insecticide application) and interspecific competition treatments. Note that y-axes are on log scales, and the y-axis of the tansy panel is gapped.

**
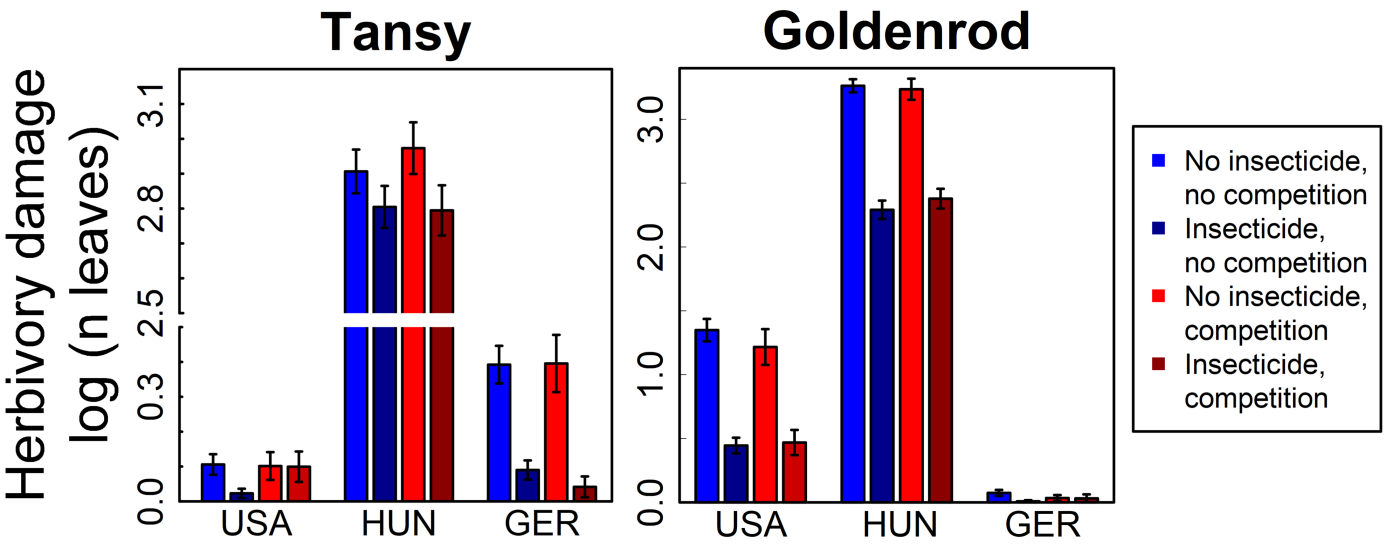
**

**Table S1.** Location, elevation, mean annual temperature (MAT; 1980-2010), and total annual precipitation (TAP) at seed collection sites. Seeds were collected in 2009.

| Country | City | Coordinates | Elevation (m) | MAT (°C) | TAP (mm) |
| --- | --- | --- | --- | --- | --- |
| Germany | Jena | 50°55´N, 11°35´E | 130 m | 9.9 °C | 610 |
| Hungary | Szentlőrinc | 46°02´N, 17°58´E | 115 m | 11.0 | 657 |
| USA | Missoula | 46°50´N, 114°02´W | 965 m | 7.7 | 359 |

**Table S2.** Locations, elevation, mean annual temperature (MAT), and total annual precipitation (TAP) for our field sites. Field experiments were conducted in 2010-2011.

| Country | City | Coordinates | Elevation (m) | MAT (°C) | TAP (mm) |
| --- | --- | --- | --- | --- | --- |
| Germany | Jena | 50°55’00N, 11°35’00E | 130 | 9.3 | 587 |
| Hungary | Pellérd | 46°01’53N, 18°08’20E | 122 | 11.0 | 675 |
| USA | Missoula | 46°50’31N, 113°59’36W | 994 | 7.7 | 359 |

**Table S3.** Locations and growing conditions for greenhouse experiments. Greenhouses were located at the University of Pecs, Hungary, and at the University of Montana, USA.

| Country | City | Day: night ratio (h) | Temp. | Rel. humidity |
| --- | --- | --- | --- | --- |
| Hungary | Pecs | 12:12 | 19.43°C | 77.5% |
| USA | Missoula | 12:12 | 19.43°C | 77.5% |

**Table S4.** Results of field experiments replicated in Germany, Hungary, and the USA that assessed the individual and joint effects of biogeographic range (native vs. non-native), country within Europe (Germany vs. Hungary), insect exclusion (insecticide), and interspecific competition on the amount of insect herbivory experienced by tansy and goldenrod, according to linear mixed-effects models with herbivore damage (number of leaves with visible insect damage on the tallest shoot of the plant; log-transformed) as the response variable; continent, country within Europe, insecticide treatment, and competition treatment as interacting fixed factors; and experimental plot nested in block (not shown) as a random factor. Significant (i.e., *P* ≤ 0.05) effects appear in bold. Numerical superscripts indicate the order in which non-significant terms (given in parentheses) were removed from the final model. Tansy and goldenrod were evaluated with independent models.

|  | Species | |
| --- | --- | --- |
| Explanatory variable | Tansy | Goldenrod |
| Range | **F_1,33_=394; *P*<<0.001** | **F_1,33_=46.6; *P*<<0.001** |
| Insecticide | **F_1,140_=20.9; *P*<<0.001** | **F_1,140_=126; *P*<<0.001** |
| Competition | (F_1,140_=0.25; *P*=0.617)^6^ | (F_1,139_=0.07; *P*=0.791)^6^ |
| Country (within Europe) | **F_1,33_=1456; *P*<<0.001** | **F_1,33_=0.01; *P*<<0.001** |
| Range × insecticide | **F_1,140_=5.03; *P*=0.026** | **F_1,140_=0.16; *P*=0.002** |
| Range × competition | (F_1,138_=0.11; *P*=0.736)^4^ | (F_1,136_=0.04; *P*=0.837)^3^ |
| Insecticide × competition | (F_1,137_=0.09; *P*=0.767)^3^ | (F_1,138_=0.65; *P*=0.422)^5^ |
| Country × insecticide | **F_1,140_=4.61; *P*=0.033** | **F_1,140_=0.05; *P*<<0.001** |
| Country × competition | (F_1,139_=0.20; *P*=0.657)^5^ | (F_1,137_=0.10; *P*=0.748)^4^ |
| Range × insecticide × competition | (F_1,136_=0.97; *P*=0.326)^2^ | (F_1,134_=0.03; *P*=0.874) ^1^ |
| Country × insecticide × competition | (F_1,135_<0.01; *P*=0.946)^1^ | (F_1,135_=0.03; *P*=0.870)^2^ |
